# Circulating Levels of Agouti-Related Peptide in Endometrial Cancer Survivors

**DOI:** 10.4021/wjon385w

**Published:** 2011-10-28

**Authors:** Julie Bienertova-Vasku, Josef Tomandl, Petr Bienert, Josef Chovanec, Zuzana Dostalova, Anna Vasku, Dalibor Valik

**Affiliations:** a Department of Pathological Physiology, Faculty of Medicine, Masaryk University, Kamenice 5 A18, Brno, Czech Republic; b Department of Biochemistry, Faculty of Medicine, Masaryk University, Kamenice 5 A16, Brno, Czech Republic; c Masaryk Memorial Cancer Institute, Brno, 602 00, Czech Republic

**Keywords:** Agouti-related peptide, Cachexia, Cancer, Cancer survivors

## Abstract

**Background:**

Recently, it has been reported that central administration of agouti-related peptide (AgRP) might have protective effect against cachexia development in tumor-bearing mice. In this study, we determined whether the disease-free endometrial cancer survivors present with different plasma AgRP levels than controls and whether there was an association with the duration of the disease-free interval.

**Methods:**

The total of 53 endometrial cancer survivors was enrolled in the study along with 93 healthy control women of similar age. Fasting blood samples were obtained and AgRP plasma levels determined using ELISA-based methodology.

**Results:**

The AgRP plasma levels were significantly higher in the cases than in the controls; AgRP levels were the lowest in obese control women (77.4 ± 19.8 pg/ml); on the contrary, the AgRP plasma levels were highest in non-obese cancer survivors (100.5 ± 21.12 pg/ml). Moreover, we observed significant differences in AgRP levels between the endometrial cancer survivors and the control subjects [p (for comparison of the cases and the controls) = 0.002]. In the regression modeling, AgRP was significantly associated with the BMI as well as the case-control status, and the case-control differences in AgRP levels retained their statistical significance also after adjustment for BMI.

**Conclusions:**

Disease-free endometrial cancer survivors who did not develop cachexia during their treatment as well as post-treatment period present with significantly higher AgRP levels than the control population, independently on their BMI and menopausal status which could be indicative of the protective effect of circulating AgRP against cachexia development in endometrial cancer.

## Introduction

Agouti related peptide (AgRP) is an endogenous antagonist of the anorexigenic neuropeptide melanocyte-stimulating hormone (MSH), derived from the proopiomelanocortin (POMC) molecule [1], that is capable of promoting food intake and positive energy balance through inhibition of the α-MSH-related signaling [2-4]. Moreover, inhibition of melanocortin signaling with AgRP or deletion of the specific receptor or treatment with melanocortin-4 receptor (MC-4R) antagonist in other experiments resulted in increased food intake and reduced energy expenditure [5-8].

The homeostasis of food intake and body weight is controlled by complex feedback loops, and even though their exact nature is largely uncertain, it is clear that hypothalamus plays a crucial role in integrating central and peripheral signaling. In the central nervous system, AgRP is produced exclusively by neurons in the arcuate nucleus (ARC) where it is co-expressed with neuropeptide Y (NPY) [9] and the inhibitory neurotransmitter γ-aminobutyric acid [10], whereas acute intracerebroventricular administration of AgRP [11] as well as overexpression of AgRP in transgenic animals [6] induce increase in body weight along with hyperphagia. This is well in accordance with the finding that the ARC is a key point integrating peripheral signals of energy balance, and regulating energy homeostasis by transmitting signals that modulate eating behavior and metabolism [12-15].

Cancer cachexia is characterized by preferential loss of adipose tissue; muscle mass is also affected [15], but wasting of muscle mass represents a common symptom of cachexia in some patients. Severe and progressive loss of fat-free mass involves both skeletal muscle and not visceral protein reserves and reflects decreases in both cellular mass and intracellular potassium concentration thus indicating severe impairment of energy metabolism [16], possibly mediated via TNF-alpha and the tumor factor proteolysis-inducing factor [17].

Loss of adipose tissue in cancer-related cachexia is secondary to a decrease in adipocyte lipid content and associates with changes in the expression of genes that regulate energy turnover, cytoskeleton and extracellular matrix, which suggests high tissue remodeling [18]. It has been proposed that changes in gene expression observed in cachexia are reciprocal to those observed in obesity [18], which implies the involvement of identical peripheral and central pathways.

Central administration of AgRP (83-132) into the lateral ventricles significantly increased body weight and food intake in tumor-bearing mice, and changes in muscle mass were similar to the tumor-free control mice [19], suggesting that AgRP may play a crucial role in regulation of energy balance in cancer-related cachexia.

The aim of the present study was to investigate differences in AgRP circulating levels between endometrial cancer survivors and healthy controls of similar age and gender distribution. We hypothesized that possible differences in circulating levels of AgRP between healthy controls subjects with various BMI and disease-free endometrial cancer survivors may exist as the cases 1) did not develop cancer cachexia; 2) mostly presented with abundant adipose tissue and 3) were obese and therefore could be expected to present with elevated leptin levels [20-21] that are inversely correlated with AgRP levels. Considering the previously reported negative correlation between AgRP plasma levels and leptin plasma levels [22], we expected the plasma AgRP levels to be negatively correlated with BMI in our study.

## Material and Methods

### Subjects

Fifty three endometrial cancer patients (mean age 63.2 ± 10.5 y) were enrolled in the study at the Clinic of Obstetrics and Gynaecology of the Masaryk University Affiliated Hospital, Brno along with 93 control women of similar BMIs and the same ethnic (Central-European Caucasian) origin (mean age 47.1 ± 12.0 y). All cases in the study were postmenopausal (mean age 63.2 ± 10.5 y); the control group consisted of 45 premenopausal women (mean age 39.2 ± 7.3 y) and 48 post-menopausal patients (mean age 57.3 ± 6.4 y).

All of the endometrial cancer cases met the following criteria: (1) diagnosed with endometrial cancer that was either confined to the uterine corpus or extended only to the cervix (FIGO [International Federation of Gynecology and Obstetrics] stage I or II (stage I, n = 31; stage II, n = 22) or SEER [Surveillance Epidemiology and End Results] stage 1- 3), (2) received treatment at the Clinic of Obstetrics and Gynaecology of the Masaryk University Affiliated Hospital, (3) treated with surgery and/or radiation, (4) age 18 years or older, (4) disease-free for three or more years (mean duration of disease-free interval was 6.52 ± 4.58 y). The diagnosis of the cancer-related cachexia was based on the criteria set in the study by Strasser *et al*. [23], whereas a weight loss of ≥ 2% or ≥ 5% within 6 months before the study that was not related to recent surgery was considered the presence of cachexia. Based on these criteria, none of the cases in the study developed cancer-related cachexia, either during the oncological therapy or after it.

The control population was recruited from healthy individuals enrolled at the Department of Preventive Medicine of the Faculty of Medicine of the Masaryk University. The presence of malignancy or severe chronic diseases was excluded by a standardized complex physical examination and personal history evaluation. The measurement of height was performed with a calibrated stadiometer and weight (in light indoor clothes and without shoes) was measured with a calibrated set of scales. The study was approved by the Committee for Ethics of Medical Experiments on Human Subjects, Faculty of Medicine, Masaryk University, Brno, and performed in adherence to the Declaration of Helsinki Guidelines. Each participant gave her written informed consent that has been archived.

### Biochemical analysis

Venous blood samples were collected into tripotassium EDTA-tubes after overnight fasting and immediately centrifuged at 1700 ≥ g for 20 min and then stored at -80 °C until analysis. AgRP plasma levels were measured using a commercially available ELISA kit (Quantikine, R&D Systems, Minneapolis, MN, USA) according to the instructions of the manufacturer.

### Statistics

Where applicable, it was first determined whether the variable presented a normal distribution using the Kolmogorov-Smirnov test, and in cases of skewed variables, logarithmic transformation was performed. For descriptive purposes, mean values are presented using untransformed values. Results are expressed as mean values and standard deviations unless otherwise stated.

In the next step, we performed Pearson’s correlation testing between AgRP and clinical as well as anthropometric parameters and we report the Pearson’s r coefficients, giving the strength of an association in the range of -1 and +1.

To identify variables that may contribute to predicting the anthropometric or clinical phenotype, we carried out a conditional logistic regression. Generally, conditional logistic regression models were used to estimate relative risks (RRs) and 95% confidence intervals (CIs) for the associations between plasma AgRP levels and endometrial cancer risk. Fisher’s exact test with Tukey-Kramer’s method of adjustment for multiple comparisons was employed for comparison of categorical variables. The data analysis was performed using the Statistica v. 8.0 (Statsoft Inc., Tulsa, OK, USA) program package. The conventional values of p ≤ 0.05 were considered statistically significant.

## Results

The baseline characteristics of the study subjects are summarized in Table 1. Overall, the mean plasma AgRP concentration was 14% higher for cases, compared to the control subjects (p = 0.002). The AgRP plasma levels were lower in non-obese control women (88.1 ± 24.4 pg/ml), and they reached their minimum in obese control women (77.4 ± 19.8 pg/ml); on the contrary, AgRP plasma level was the highest in non-obese endometrial cancer survivors (100.5 ± 21.12 pg/ml) and slightly lower in obese endometrial cancer survivors (91.5 ± 33.1 pg/ml); the differences in AgRP between investigated groups were statistically significant [p (for comparison of the cases and the controls) = 0.002]. The mean AgRP plasma concentrations remained consistently higher among cases, compared with the controls, when stratified by menopausal status, BMI or smoking status, although the differences were not always statistically significant. In a separate analysis of AgRP plasma levels according to parity, no significant associations were observed either in the cases or the controls (Table 2). No association of AgRP plasma levels with age was observed (r = -0.015, p = 0.858).

**Table 1:** Baseline Characteristics of the Study Subjects

Baseline characteristics	Cases	Controls	p-value
Total number	53	93	
Menopausal status			
Premenopausal	-	45 (48%)	-
Postmenopausal	53 (100%)	48 (52%)	0.008
Age (yr)	63.2 ± 10.5	47.1 ± 12.0	≤0.0001
Age at diagnoses (yr)	56.6 ± 10.4	-	-
Age at menarche	13.3 ± 1.4	12.9 ± 1.6	NS
Age at menopause	50 ± 5.3	47.6 ± 5.4	0.006
BMI [kg.m^-2^]	32.0 ± 6	36.1 ± 11.8	0.06
Parity	1.8 ± 1.1	1.4 ± 1	NS
History of spontaneous miscarriage	9 (17%)	18 (19%)	NS
Endometrium / myometrium index (EMI)	18 ± 9.2 / 40.7 ± 10.1	-	-
Grading	1.7 ± 0.8	-	-
Length of disease-free period	6.5 ± 4.6	-	-
Self reported diabetes (%)	34.0%	10.8%	0.005
Plasma AgRP levels (pg/ml)			
All women	94.7 ± 29.5	81.5 ± 22.2	0.002
Non-obese (BMI < 30 kg/m^2^)	100.5 ± 21.12	88.1 ± 24.4	0.008
Obese (BMI ≥ 30 kg/m^2^)	91.5 ± 33.1	77.4 ± 19.8	0.03

Values given as mean ± SD, p = differences between the cases and controls analysed using Kruskal-Wallis test and Fisher’s exact test

**Table 2:** Plasma AgRP Values According to the Parity

Parity	0	1	2	≥ 3	p
Cases	93.4 ± 30.4	93.6 ± 36.5	98 ± 30.4	89.6 ± 21	0.96
Controls	84.3 ± 15.1	80.8 ± 29.7	79.2 ± 21.1	86.2 ± 27.9	0.39

Values given as mean ± SD, p = differences between the cases and controls analysed using Kruskal-Wallis test and Fisher’s exact test

### AgRP plasma levels and obesity

Pearson partial correlation coefficients, adjusted for age and case-control status (i.e. in analysis performed across the pooled survivors and control subjects), showed that plasma AgRP levels were significantly negatively correlated with investigated measures of adiposity. Conditional logistic regression analyses revealed a strong association between AgRP concentration and case-control status (β = -0.22, p = 0.006), independently on BMI of the subjects.

When performing the univariate regression tests across the whole studied cohort (pooled cases and the controls), AgRP expressed correlation with BMI (β = -0.22, p = 0.006). This observed relationship retained its statistical significance also after adjustment for age and smoking status (β = -0.22, p = 0.006), whereas the non-obese females presented with higher levels of AgRP (92.4 ± 23.9 pg/ml) than the obese women (82.7 ± 26.3 pg/ml; p = 0.009).

### AgRP plasma levels according to menopausal status

AgRP plasma levels in premenopausal control women (83.8 ± 24.1 pg/ml) and postmenopausal control women (79.4 ± 20.2 pg/ml) tended to be lower than in the cases that were all postmenopausal (94.7 ± 29.5 pg/ml; respectively, p = 0.40), however, these results did not achieve statistical significance. No association of AgRP plasma levels with age at menarche or age at menopause in the controls was observed; the cases were excluded from this analysis as menopause in some of the cases resulted artificially from the treatment of the disease.

## Discussion

In the presented study, we investigated plasma AgRP levels in a sample of 53 endometrial cancer survivors and compared them to 93 controls of similar age, BMI and various menopausal status. We demonstrated significant differences in AgRP plasma levels between obese and non-obese endometrial cancer survivors as well as across the whole cohort of endometrial cancer survivors and the controls, whereas this association was age-independent. Moreover, we observed a significant association of AgRP levels previous history of endometrial cancer, independently on BMI and age, indicating that endometrial cancer survivors could present with habitually elevated AgRP levels compared to the control population that could protect them against cachexia development.

Physiological plasma levels of AgRP show significant variation during life, the average plasma levels reported for the adolescent girls with BMI are 30.1 ± 16.9 pg/ml [24], while Katsuka *et al*. [25] report significant differences in AgRP levels between the obese (14.46 ± 2.5 pg/ml) and nonobese women (6.0 ± 0.5 pg/ml, p < 0.05), hence on a very small population sample. In another study, reported plasma levels of AgRP were 49.4 ± 2.4 pg/ml in the obese individuals compared to 10.1 ± 0.9 pg/ ml in the lean subjects and there was a significant correlation with the fat mass (P < 0.001), percentage body fat (P < 0.001) and leptin (P < 0.05) [22]. The AgRP levels observed in our were almost 2-times higher in our study, but it has to be mentioned that the study by Hoggard *et al.* was conducted on a very small set of male volunteers and no large population study is available so far to estimate gender-related effects of AgRP. Recently, it has been reported by our research group that plasma levels of AgRP express extreme variation both in maternal serum and umbilical cord blood in the peripartum period [26], which is indicative of AgRP plasma levels showing substantial variations during life. The differences in AgRP levels between the presented study and other published papers could be also explained by the effect of age as our study subjects were significantly older. The other possible explanation could be that the observed higher AgRP levels were associated with higher BMI of our study subjects, which is also consistent with our finding of significant correlation of BMI and AgRP circulating levels.

Our pilot results showed that AgRP plasma levels differ between endometrial cancer survivors and healthy population; although this correlation was BMI-dependent, it retained the statistical significance after adjustment for BMI; the non-obese women with long-term disease-free survival of endometrial cancer presented with highest AgRP levels, while the lowest AgRP levels were observed in obese healthy women.

It has been known for a long time that successful removal of the tumor can be associated with the cachexia reversal process and significant changes in various metabolic pathways were observed after tumor excision in experimental animals [27-28]. It has to be noted, however, that the patient selected for our study were completely disease-free for at least three years, so we did not expect any significant effect of tumor removal on the actual AgRP levels in these subjects and we presume that the increased AgRP levels are rather a constitutive trait or a function of BMI than a reaction to tumor removal.

The exact nature of the regulatory feedback circuits involving AgRP, regulating feeding and satiety behavior remains unclear. The previous reports on AgRP plasma levels in relation to BMI are highly controversial. It has been reported that plasma levels of AgRP were elevated in anorexia nervosa [29], while plasma alpha-MSH levels were not significantly different in this study. On the other hand, elevated levels of AgRP were observed in a cohort of obese men, where the AgRP levels were correlated with the plasma levels of alpha-MSH, independently from the total fat area [25]. In our study, we observed increased AgRP levels in non-obese subjects, independently on their case-control status. The inverse relationship between AgRP and BMI could be explained by previously reported inverse relationship between AgRP plasma levels and plasma leptin levels [22]. Indeed, plasma AgRP levels were experimentally suppressed by peripheral leptin administration in fasted humans [24].

As we observed a strong trend towards the increased AgRP levels in endometrial-cancer survivors, which remained significant also after adjustment for BMI, we hypothesized that apart from possible relationship of AgRP levels and BMI, reflecting roughly the fat “reserves”, there may be another underlying mechanism making the cancer survivors in our study less prone to develop cancer cachexia than others. It is possible that the increased circulating AgRP levels might stimulate feeding behavior and inhibit pro-cachechtic pathways, thus making our cancer survivors less prone to cachexia-related complications. Our observation is well in accordance with the findings on animal models by Markison *et al*. [30-31] reporting that central application of exogenous AgRP (83-132) or systemic MC4R antagonists can protect against the anorexic effect of LLC (Lewis lung carcinoma) tumors.

Taken together, our data suggest that AgRP may play a role in preventing cancer-related cachexia development, which supports the hypothesis of possible clinical utility of AgRP agonists. However, further studies focused on potential therapeutic potential of AgRP agonists on substantially larger population samples with different ethnicity and age structure, possibly with prospective design, are a necessity.
